# Ginseng Polysaccharides Inhibit *Aspergillus sydowii*-Driven Lung Adenocarcinoma via Modulating Gut Microbiota–Bile Acid Metabolism Axis

**DOI:** 10.3390/cancers17193134

**Published:** 2025-09-26

**Authors:** Jinlian He, Xiao Shu, Hudan Pan, Mingming Wang, Yuanyuan Song, Feng Zhou, Lirong Lian, Liqing Chen, Gangyuan Ma, Yicheng Zhao, Runze Li, Liang Liu

**Affiliations:** 1 Guangzhou National Laboratory, Guangzhou 510000, China; 2 State Key Laboratory of Traditional Chinese Medicine Syndrome/Chinese Medicine, Guangdong Laboratory, The Second Affiliated Hospital of Guangzhou University of Chinese Medicine, Guangzhou 510006, China; 3 Institute of Traditional Chinese Medicine Pharmacology, Shandong Academy of Chinese Medicine, Jinan 250014, China

**Keywords:** lung adenocarcinoma (LUAD), Ginseng Polysaccharides (GPs), *Aspergillus sydowii*, gut microbiota, bile acid metabolism

## Abstract

Background: Lung cancer is the leading cause of cancer-related mortality globally, with lung adenocarcinoma (LUAD) as the most common subtype. Dysbiotic intratumoral mycobiomes drive LUAD pathogenesis, and *Aspergillus sydowii* (*A. sydowii*) acts as a key oncogenic fungal species. Ginseng polysaccharides (GPs), bioactive phytochemicals with immunomodulatory and oncostatic properties, counteract fungal infections and restore immunosurveillance in LUAD. Methods: Subcutaneous and orthotopic LUAD murine models were established by implanting Lewis lung carcinoma (LLC) cells. Subcutaneous tumors were infected intratumorally and orthotopic models via nasal inoculation. GPs (200 mg/kg/day) were orally administered to evaluate tumor growth. Metagenomic and targeted bile acid metabolomic profiling of fecal and tumor tissues was performed, with Spearman correlations analyzed using R packages. Results: GPs significantly inhibited *A. sydowii*-induced tumor growth in both models. In subcutaneous tumors; GPs reduced volume (*p* < 0.05) and weight vs. infected controls. In orthotopic models, GPs decreased pathological nodules and lung weight, with micro-CT/H&E confirming attenuated hyperplasia. Metagenomics showed GPs restored gut homeostasis by enriching *Lactobacillus/Muribaculum intestinale* and suppressing pro-inflammatory *Alistipes*. Targeted metabolomics revealed reduced β-Hyodeoxycholic Acid (3β-HDCA), Chenodeoxycholic acid 24-acyl-b-D-glucuronide (CDCA-24G) and 3β-hydroxychol-5-en-24-oic acid (5-isoLCA) after GP treatment. Network analysis confirmed significant microbe–bile acid interactions. Conclusions: GPs exert antitumor effects against *A. sydowii*-induced LUAD by modulating gut microbiota and bile acid metabolism. This identifies GPs as a promising therapy for mycobiome-influenced cancers, with dual targeting of fungal infection and metabolic reprogramming.

## Simple Summary

Emerging evidence implicates the intratumoral mycobiome in lung cancer progression. Here, we demonstrate that the oncogenic fungus *Aspergillus sydowii* accelerates tumor growth in a murine model of Lewis lung carcinoma. Ginseng polysaccharides (GPs) counteracted these effects by remodeling microbial communities in the gut and lung. Specifically, GPs restored populations of beneficial bacteria while reducing pro-inflammatory pathogens and decreasing levels of carcinogenic bile acids derived from *A. sydowii*. Integrated metagenomic and metabolomic analyses revealed that GPs modulate the microbiota–bile acid axis, thereby attenuating fungal-driven tumorigenesis. These results underscore the therapeutic potential of targeting host–microbe interactions with natural compounds like GPs to suppress mycobiome-associated lung cancer.

## 1. Introduction

Lung cancer remains the leading cause of cancer-related mortality globally, claiming approximately 350 lives daily, with LUAD constituting the most prevalent histological subtype [1]. The interplay between microbial communities and human malignancies has emerged as a focal research area in recent years. Notably, dysbiotic microbiomes have been identified as a hallmark of malignancy, exerting profound impacts on tumorigenesis and treatment efficacy [2]. While the lung was traditionally considered a sterile environment, the Human Microbiome Project demonstrated the presence of resident microbial communities. Analogous to the gut microbiome, the intratumoral mycobiome has gained traction for its potential diagnostic and prognostic utility in cancer management [3].

Concurrently, the role of microbial communities in oncogenesis has become a research hotspot. Accumulating evidence underscores the pivotal role of fungal infections in lung cancer initiation and progression. For instant, *Candida albicans*, as a common opportunistic pathogen, disrupts pulmonary architecture and function through toxin/enzymatic secretion, thereby fostering tumor cell proliferation and invasion [4]. *Aspergillus fumigatus* can activate the inflammatory signaling pathway in the lung, resulting in the formation of a chronic inflammatory microenvironment and providing suitable soil for the occurrence and development of tumors [5].

Recent studies have identified *A*. *sydowii* as a core constituent of the LUAD intratumoral mycobiome, with its abundance inversely correlating with patient survival [6]. However, managing these infections presents multiple challenges: firstly, the low biomass of intratumoral fungi complicating early detection via conventional methods [7]; secondly, inherent resistance of fungi to traditional antifungal agents (e.g., amphotericin B, voriconazole) [8]; and thirdly, the combined use of chemotherapy or immunosuppressants potentially further compromising host defenses [9]. Thus, there is an urgent need for novel therapeutic strategies that can simultaneously target fungal infections and reverse immunosuppression.

Ginseng polysaccharides (GPs) have emerged as promising phytochemical candidates due to their dual immunomodulatory and oncostatic capabilities, particularly through macrophage polarization and dendritic cell maturation [10]. Notably, in fungal-driven pulmonary pathologies, GPs mitigate IL-17-driven neutrophilic inflammation through bidirectional regulation of the gut–lung axis, mediated by probiotic Lactobacillus enrichment and pathogen exclusion [11]. In the present study, Lewis’s carcinoma-bearing C57BL/6 mice with subcutaneous tumors and orthotopically implanted lung malignancies were employed to systematically interrogate the mechanistic basis of GPs against *A*. *sydowii*-associated lung carcinogenesis. Moreover, a microbiota–gut metabolomics study was performed to clarify the possible mechanisms by which GPs provided protection against lung cancer caused by *A*. *sydowii*.

## 2. Materials and Methods

### 2.1. Lewis Lung Carcinoma Murine Model

C57BL/6J mice were subcutaneously implanted with 1 × 10^6^ LLC cells suspended in Matrigel (Corning, 354248, New York, NY, USA) to establish ectopic neoplasia. Tumor volumes were measured triweekly using digital calipers (length × width^2^ × 0.5) to monitor growth kinetics. Following tumor establishment (100 mm^3^), tumors were injected with 1 × 10^7^ CFU/mouse *A. sydowii* in 25 μL PBS via intratumoral injection to model mycobiota-driven oncogenesis. The *A. sydowii* (ID: 3A00237) strain was obtained from the China Ocean Microbial Strain Conservation and Management Center (MCCC, Xiamen, China), which was originally isolated from deep-sea sediments. The GP treatment group received oral administration of 200 mg/kg GPs (Lot No. MUST-23112303, MUST, Chengdu, China) from a reputable supplier of natural products and biochemical reagents, and the certificate of analysis confirmed a purity of ≥98% (w/w) via UV spectrophotometry. Host–pathogen interactions were monitored via metabolic parameters including food intake, body weight changes, and hydration status.

### 2.2. Orthotopic LUAD Murine Model

Orthotopic LUAD murine models were established by intratracheal inoculation of 1 × 10^6^ LLC cells in C57BL/6J mice. To model fungal–tumor interactions, mice received intratracheal *A. sydowii* challenges (1 × 10^7^ CFU/mouse in 25 μL PBS) every three weeks for four cycles. Concurrently, the GP group received oral administration of 200 mg/kg GPs to assess its impact on pulmonary colonization until ethical euthanasia endpoints. Host–pathogen interactions were monitored via metabolic parameters such as food intake, body weight fluctuations, and hydration status.

### 2.3. Metagenomics Analysis of Gut Microbiota

Metagenomic DNA was extracted from Lewis lung carcinoma mice using the QIAamp DNA Stool Mini Kit (QIAGEN, Hilden, Germany), followed by library construction with the NEBNext Ultra DNA Library Prep Kit (New England Biolabs, Ipswich, MA, USA). Shotgun metagenomic sequencing was performed on the [MGISEQ-2000/DNBSEQ-T7] platform (BGI, Beijing, China) using a paired-end 150 bp (PE150) strategy (Beijing, China).

Raw data were processed using SOAPnuke for quality control and Bowtie2 for host read depletion. Filtered reads were assembled de novo using MEGAHIT with k-mer optimization, generating contigs for open reading frame prediction via MetaGeneMark. A non-redundant gene catalog was constructed using CD-HIT (90% identity threshold), and gene abundances were quantified via Salmon alignment. Functional annotation was conducted using DIAMOND/RGI [12,13] against specialized databases (e.g., NOG, KEGG, BacMet, CARD, COG, CAZy, Swiss-Prot) to characterize metabolic pathways and antimicrobial resistance profiles. Taxonomic profiling was performed using Kraken2 [14] with custom-curated reference databases (NCBI NT/UHGG), followed by abundance refinement with Bracken2 to characterize mycobiota and bacterial community dynamics at the species level.

Alpha-diversity, representing the within-sample microbial richness and evenness, was assessed using the Shannon index and the observed species (richness) metric. These indices were calculated from the species-level abundance table using the ‘vegan’ package (v2.6-4) in R. Differences in alpha-diversity indices between experimental groups were compared using the Kruskal–Wallis test followed by Dunn’s post hoc test, as the data did not meet the assumptions of parametric testing.

Beta-diversity analysis was conducted to assess the compositional differences between microbial communities. A species-level abundance table was generated and used to calculate the Bray–Curtis dissimilarity matrix using the ‘vegdist’ function in the R package ‘vegan’ (v2.6-4). Principal coordinate analysis (PCoA) was initially performed to visualize the overall clustering patterns. Subsequently, partial least squares discriminant analysis (PLS-DA), a supervised method designed to maximize the separation between predefined groups, was employed using the ‘plsda’ function in the R package ‘mixOmics’ (v6.22.0). The PLS-DA model was validated using a 10-fold cross-validation approach to assess its predictive accuracy and prevent overfitting. The resulting dissimilarity matrices and ordination plots were used to visualize the clustering of samples based on their microbial community profiles.

### 2.4. Targeted Metabolomic Analysis of Bile Acids

Targeted bile acid (BA) metabolomic profiling was performed on an LC-MS/MS system. Chromatographic separation was achieved using a Waters ACQUITY UPLC HSS T3 C18 column (1.8 µm, 100 mm × 2.1 mm i.d.) maintained at 40 °C on an ExionLC™ AD system (Sciex). The mobile phase consisted of (A) ultrapure water containing 0.01% acetic acid and 5 mM ammonium acetate and (B) acetonitrile containing 0.01% acetic acid. The following gradient elution program was used at a flow rate of 0.35 mL/min: 0–2 min, 20% B; 2–12 min, 20–80% B; 12–13 min, 80–100% B; 13–15 min, 100% B; 15–15.1 min, 100–20% B; 2.9 min re-equilibration period at 20% B. The injection volume was 3 µL. For precise quantification, a comprehensive set of authentic standards—including UDCA-3S, DCA-3-O-S, HCA, MDCA, 3-oxo-CA, 3β-UDCA, UDCA, CA, 3β-HDCA and HDCA—was used to construct analyte-specific calibration curves. Serial dilutions of these standards were prepared across a wide dynamic range (0.1–4000 ng/mL) at 15 concentration levels (0.1, 0.2, 0.4, 1, 2, 4, 10, 20, 40, 100, 200, 400, 1000, 2000, and 4000 ng/mL). CDCA-d4 was used as the internal standard to correct for sample preparation and ionization variability. Data were acquired via multiple reaction monitoring (MRM) in ESI-negative mode. Briefly, 20 mg of tumor tissue was weighed, proteins were precipitated, and samples were lyophilized. Dried samples were redissolved and centrifuged, after which 5 μL of supernatant was transferred to a 96-well plate for LC-MS analysis. Raw LC-MS data were processed with MultiQuant 3.0.3 software for peak integration, calibration, and metabolite quantification. Linearity Validation Calibration curves for 82 bile acids were constructed using 15 concentration levels of authentic standards (0.1–4000 ng/mL), including UDCA-3S, DCA-3-O-S, HCA, MDCA, 3-oxo-CA, 3β-UDCA, UDCA, CA, 3β-HDCA, and HDCA. Linear regression was performed by plotting the peak area ratio (analyte/CDCA-d4) against nominal concentration, with a weighting factor of 1/x^2^. Precision was evaluated using QC samples (low: 0.4 ng/mL; medium: 20 ng/mL; high: 400 ng/mL) and validated via Pearson correlation analysis and coefficient of variation (CV) analysis. Statistical analyses were conducted using GraphPad Prism 9.0 (San Diego, CA, USA).

### 2.5. Correlation of Metagenomics-Targeted Metabolomic Analysis of Bile Acids

Canonical correspondence analysis (CCA) was performed using the CCA package in R to examine associations between differentially abundant metabolites and microbial taxa across taxonomic levels. This approach utilized composite variables to quantify the integrative association between microbial communities and bile acid metabolism. Pearson and Spearman correlation analyses were conducted using the cor function in R, based on metabolite and microbial species abundance matrices. The screening criteria were |r| ≥ 0.6 and *p* < 0.05. Visualization of significant correlations was achieved using ggplot2 or heatmap packages, with heatmaps and Circos plots employed to illustrate metabolite–microbe associations.

### 2.6. Association Between Metabolomics Data and Clinical Outcome

An integrative cross-study metabolomic analysis was performed to explore the association between gut microbiota, bile acid metabolites, and LUAD risk. Gut microbiota data from lung cancer patients (LC, *n* = 35, 18–50 years) and healthy controls (HC, *n* = 18) were obtained from Wang et al. [15]. Gut microbiota profiling at the genus and species levels was analyzed using the R package ComplexHeatmap. Following data normalization, hierarchical clustering based on Euclidean distance was performed to evaluate microbial community structure and taxonomic abundance differences between groups.

Moreover, plasma metabolite data from NSCLC patients (NSCLC, *n* = 16, 18–50 years) and healthy controls (HC, *n* = 8) were obtained from the Mendeley Data repository (Ni, Boxiong, 2023 [16]). Hierarchical clustering analysis of bile acid metabolites was conducted using the R package Complex Heatmap. Raw data were subjected to log10 (x + 1) transformation, followed by clustering analysis with Euclidean distance.

### 2.7. Statistical Analysis

All data are presented as mean ± standard deviation (SD). The normality of distribution was assessed using the Shapiro–Wilk test, and the homogeneity of variances was assessed using the Brown–Forsythe test. For data that met both assumptions, statistical significance was determined using one-way ANOVA followed by Dunnett’s test for multiple comparisons. For data that did not meet the assumptions of parametric testing, the non-parametric Kruskal–Wallis test followed by Dunn’s test was applied. A *p* value < 0.05 was considered statistically significant. All analyses were performed using GraphPad Prism 9.0 (San Diego, CA, USA).

## 3. Result

### 3.1. GPs Attenuate Neoplastic Proliferation and A. sydowii-Driven LUAD Carcinogenesis

**Figure 1:**
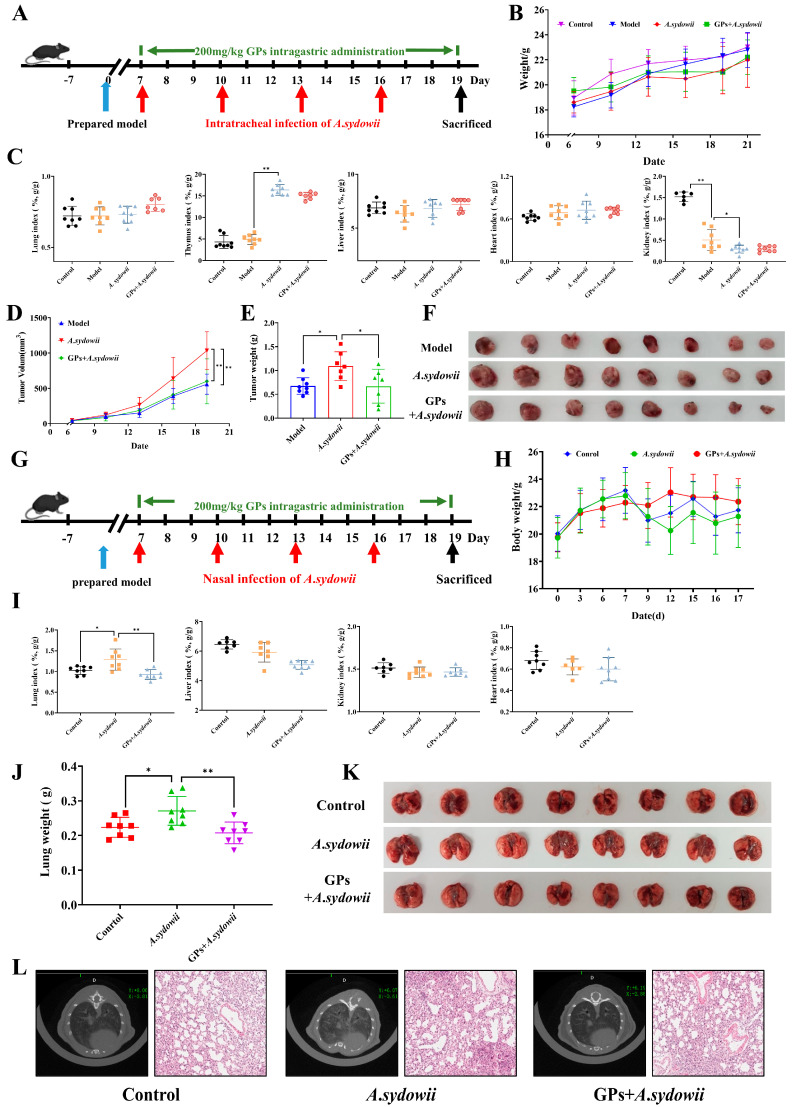
GPs suppress neoplastic progression. (**A**) Protocol for GPs’ impact on *A*. *sydowii*-infected Lewis mice. (**B**) The body weight of Lewis mice. (**C**) The organ coefficient of Lewis mice. (**D**) The tumor volume variation of Lewis mice. (**E**) The tumor weight of Lewis mice. (**F**) Tumor in Lewis Mice. (**G**) Protocol for GPs’ impact on lung lesions in *A*. *sydowii*-infected orthotopic LUAD mice. (**H**) The body weight of orthotopic LUAD mice. (**I**) The organ coefficient of orthotopic LUAD mice. (**J**) Lung weight in orthotopic LUAD mice. (**K**) Effect of GPs on lung lesions in *A*. *sydowii*-infected orthotopic LUAD mice. (**L**) Micro-CT and H&E images of lung tissue in orthotopic LUAD mice. Data were expressed as mean ± SD. Significant comparison was determined by the Kruskal–Wallis test followed by Dunn’s multiple comparisons test (or one-way ANOVA with Dunnett’s test if the data passed normality and homogeneity tests); *n* = 8, * p < 0.05, ** p < 0.01 vs. *A*. *sydowii* group.

To determine if GPs could counteract the effects of *A. sydowii* in promoting LUAD tumor growth, *A. sydowii* was injected intratumorally in a Lewis lung cancer mouse model. Meanwhile, the mice received GPs orally at a daily dosage of 200 mg/kg to evaluate the impact of GPs on subcutaneous tumor growth (Figure 1A). We found that unlike the bodyweight organ index and routine blood test (Figure 1B,C and Figure S1), *A. sydowii* significantly enhanced the growth of subcutaneous tumors in the Lewis mice compared to the control group, while the subcutaneous tumor growth was significantly suppressed in the GP group (Figure 1F). Moreover, there were also significant differences in the inhibitory effects on the volume and weight of the subcutaneous tumors in mice (Figure 1D,E).

Moreover, to validate the findings previously reported, we developed an orthotopic LUAD murine model that more accurately mimics the LUAD microenvironment. This was achieved by administering LLC cells into C57BL/6J mice through the tail vein (Figure 1G). We verified the successful induction of tumors within the lung parenchyma, and although the *A*. *sydowii* infection did not significantly affect the body weight, organ index and routine blood test of the mice (Figure 1H,I and Figure S2), it did accelerate pathological changes in the lung tissues of the orthotopic lung cancer model compared to the control group. Following the oral administration of GPs, there was a reduction in the number of pathological nodules on the lung tissue surface and a decrease in lung tissue weight (Figure 1J,K). Additionally, the H&E staining and micro-CT imaging demonstrated that hyperplasia occurred in the lung tissues post tail vein injection of LLC cells, with pronounced abnormal hyperplasia following *A. sydowii* infection; however, GPs were found to significantly inhibit this abnormal hyperplasia (Figure 1L).

### 3.2. GPs Modulate Gut Microbiota Diversity in A. sydowii-Challenged Lewis Mice

**Figure 2:**
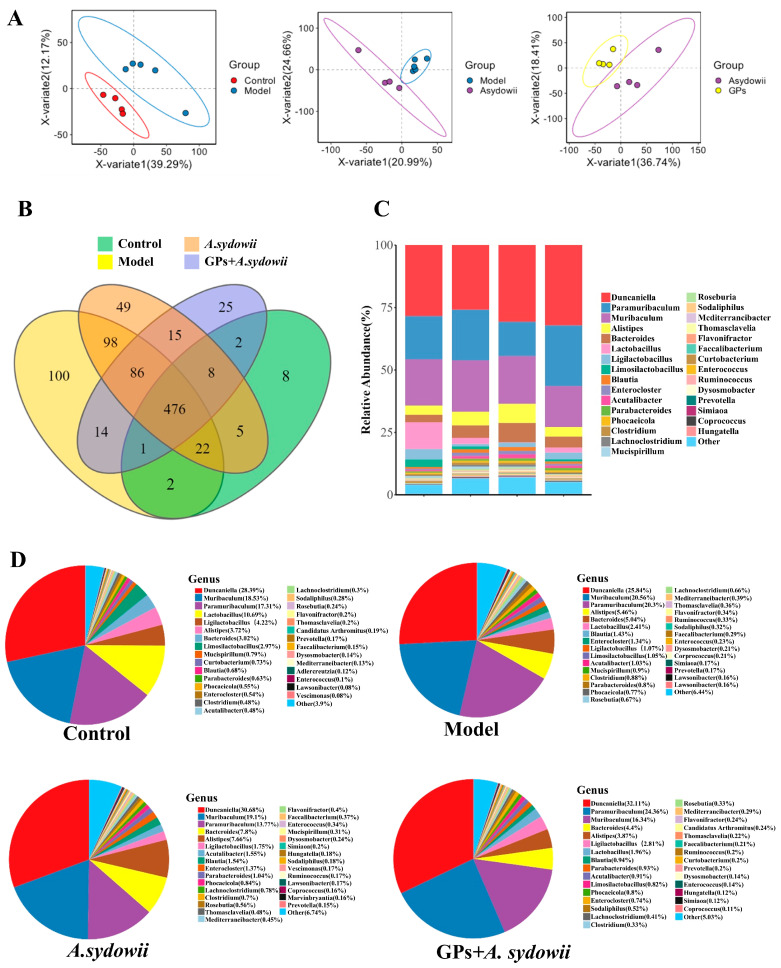
Delineating the taxonomic–functional interplay in Lewis murine gut microbiota through metagenomic profiling. (**A**) PLS-DA of β diversity difference analysis. (**B**) OTU Venn diagram of genus level. (**C**) The structure of the community among groups at the genus level; (**D**) relative abundances of gut microbiota at the genus level, *n* = 5.

To evaluate GPs’ impact on gut microbiota richness, diversity, evenness, and clustering, we conducted species diversity analysis. We used β diversity analysis PLS-DA to reflect gut microbiota clustering. The symbols for the two mouse groups formed group-based clustering patterns in both analyses’ score plots, though some overlap was observed (Figure 2A and Figure S3), indicating mild differences in fecal microbiota structure between the groups.

Then we analyzed fecal microbiota compositions at the genus level. All four groups shared 476 OTU numbers. The *A. sydowii* infection group had a relatively high proportion of lung-specific microbiota, while the control and GP + *A. sydowii* groups had lower proportions (Figure 2B). We detected 30 phyla in all mice’s lung microbiota (excluding unnamed phyla). At the genus level, the predominant bacteria across the five groups were *Duncaniella*, *Paramuribaculum*, *Muribaculum, Alistipes*, and *Bacteroides* (Figure 2C,D).

### 3.3. GPs Alleviate Gut Microbiota Dysbiosis in A. sydowii-Infected Lewis Mice

**Figure 3:**
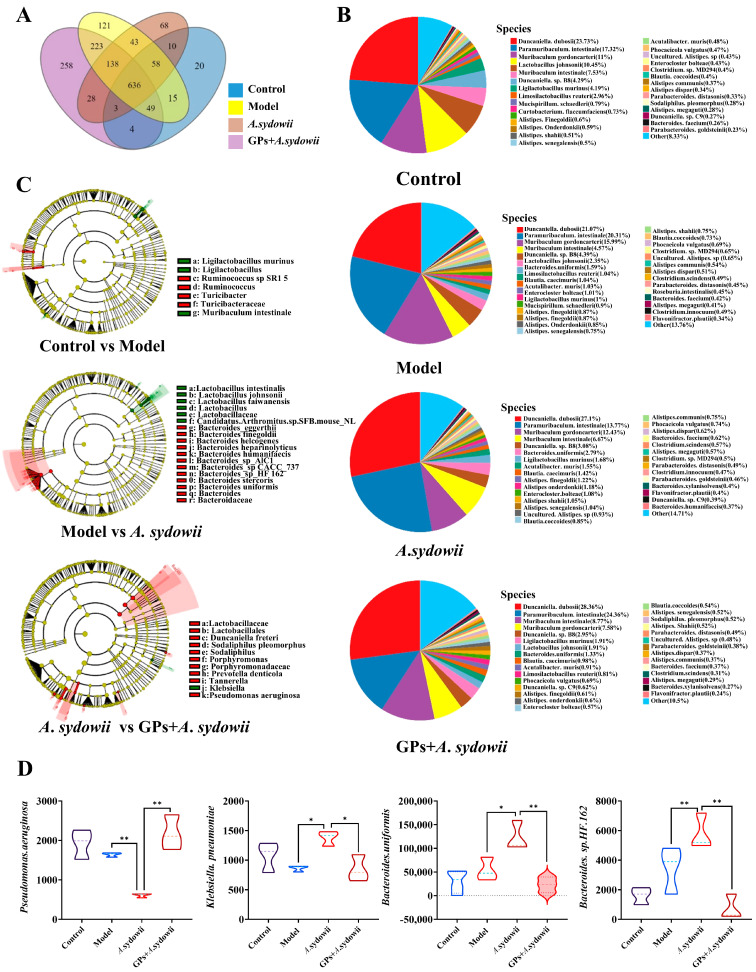
The differential microbiota in *A*. *sydowii*-infected Lewis mice. (**A**) OTU Venn diagram of species level; (**B**) relative abundances of gut microbiota at the species level; (**C**) LEfSe multi-level branching tree map; (**D**) differential gut microbiota ai species level. Data were expressed as mean ± SD. Significant comparison was determined by the Kruskal–Wallis test followed by Dunn’s multiple comparisons test (or one-way ANOVA with Dunnett’s test if the data passed normality and homogeneity tests); *n* = 5, * p < 0.05, ** p < 0.01 vs. *A*. *sydowii* group.

Species-resolved metagenomic profiling was performed across disease progression stages to characterize temporal dynamics of fecal microbiota during *A. sydowii*-driven pulmonary oncogenesis. Similar to genus-level analysis, all four groups shared 636 operational taxonomic units (OTUs), with the model group exhibiting the highest proportion of lung-specific microbiota (Figure 3A). Gut microbiota composition in the GP group was significantly more similar to the control group, with marked elevation in *Paramuribaculum intestinale*, *Muribaculum intestinale*, *Ligilactobacillus murinus*, and *Lactobacillus johnsonii*, and reduced *Muribaculum gordoncarteri* (Figure 3B).

LEfSe and variance analyses were performed to identify GP-responsive gut bacteria. LEfSe multi-level cladograms revealed taxonomic differences across phylum-to-species levels among the four groups. Differential gut microbiota was identified using log_2_ (fold change) >1 and *p* < 0.05 as thresholds, including *Guncaniella freteri, Sodaliphilus pleomorphus, Prevotella denticola, Pseudomonas aeruginosa*, and *Klebsiella spp*. (Figure 3C). GP treatment increased *Pseudomonas aeruginosa* and decreased *Klebsiella pneumoniae*, *Bacteroides uniformis*, and *Bacteroides* sp. HF.162 compared to *A. sydowii*-infected mice (Figure 3D and Figure S4).

### 3.4. GPs Modulate Bile Acid Metabolism Disrupted by A. sydowii Infection in Lung Cancer

**Figure 4:**
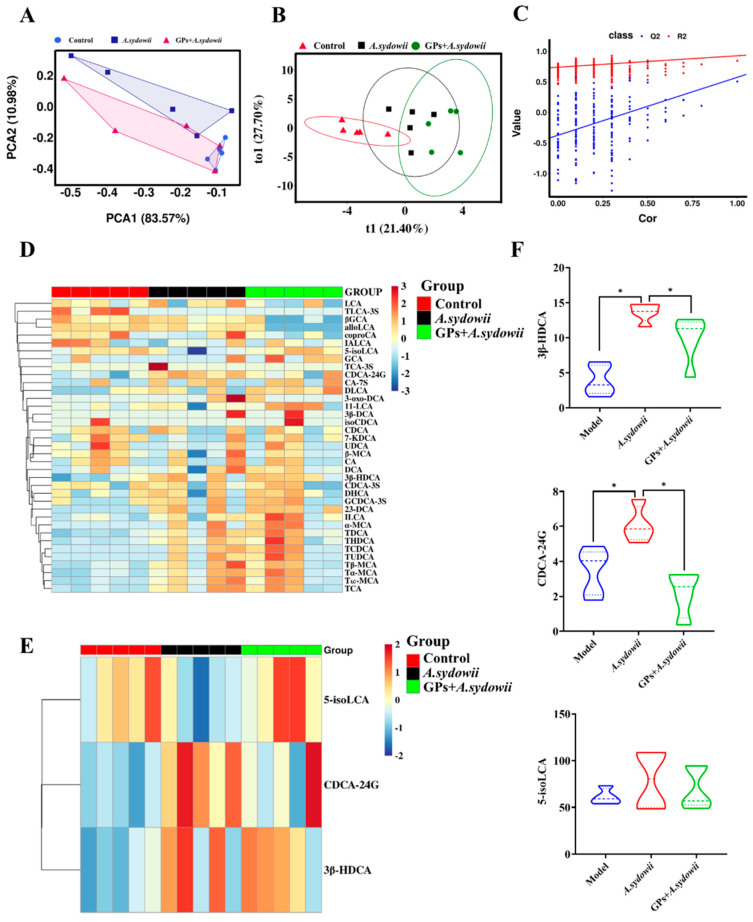
Restoration of bile acid metabolic homeostasis in A. *sydowii*-infected lung cancer model. (**A**) PCA score plot; (**B**) OPLS-DA score plots derived from tumor tissue; (**C**) validation plot of OPLS-DA model; (**D**) heatmap of all bile acid metabolites across different groups; (**E**) heatmap of differentially expressed bile acid metabolites; (**F**) quantitative analysis of differential metabolites in each group. Data were expressed as mean ± SD. Significant comparison was determined by the Kruskal–Wallis test followed by Dunn’s multiple comparisons test (or one-way ANOVA with Dunnett’s test if the data passed normality and homogeneity tests); *n* = 5, * p < 0.05.

To investigate altered bile acid metabolites in *A*. *sydowii*-infected Lewis mice with GP administration, we performed targeted bile acid metabolomic mass spectrometry analysis of tumor tissues, which included a total of 82 bile acid metabolism-related metabolites (Table S1). Matrix effects were evaluated using QC samples to ensure the reliability and accuracy of our LC-MS/MS method. The Pearson correlation coefficients (|r|) between QC replicates were calculated to assess the consistency of detection signals. The results showed high correlation coefficients of 0.9937, 0.9956, and 0.9963 for low-, medium-, and high-concentration QC samples. And the ECDF of CV values for the metabolites showed that over 80% of the metabolites had a CV below 0.3 and more than 80% had a CV below 0.2, confirming the high precision and low variability of our measurements. Respectively, these results indicate excellent system stability and minimal matrix effects (Figure S5). Principal component analysis (PCA) revealed significant differences in the cluster analysis of bile acid metabolites between the model group, *A. sydowii* group and GP + *A*. *sydowii* group (Figure 4A), and the differences between the groups were statistically significant (*p*= 0.024, *r* = 0.3), indicating that *A. sydowii* infection significantly altered the host’s bile acid metabolic profile, and GP intervention might have a certain regulatory effect on this metabolic disorder.

To further explore potential biomarkers, OPLS-DA was established for clustering (Figure 4B). The results showed significant metabolic differences between the model control group and the *A. sydowii*-infected group, and the model’s goodness of fit (R^2^ = 0.7384) indicated that the model had good reliability (Figure 4C). Through heatmap analysis of differential metabolites (Figure 4D,E), we found that *A. sydowii* infection led to significant changes in a variety of bile acid metabolites, and GP intervention reversed the abnormal expression of these metabolites to a certain extent. The quantitative analysis results (Figure 4F and Figure S6) further confirmed that compared with the model control group, the levels of bile acid metabolites such as 3β-HDCA, CDCA-24G, and 5-isoLCA in the *A. sydowii*-infected group changed significantly, while the combined treatment with GPs adjusted the levels of these metabolites back to the direction of the model control group, suggesting that ginseng polysaccharide may exert its therapeutic effect on lung cancer caused by *A. sydowii* infection by regulating bile acid metabolism.

### 3.5. Comparative Assessment Unveiling Distinctions in Gut Microbiome Composition and Metabolic Profiles in LUAD

**Figure 5:**
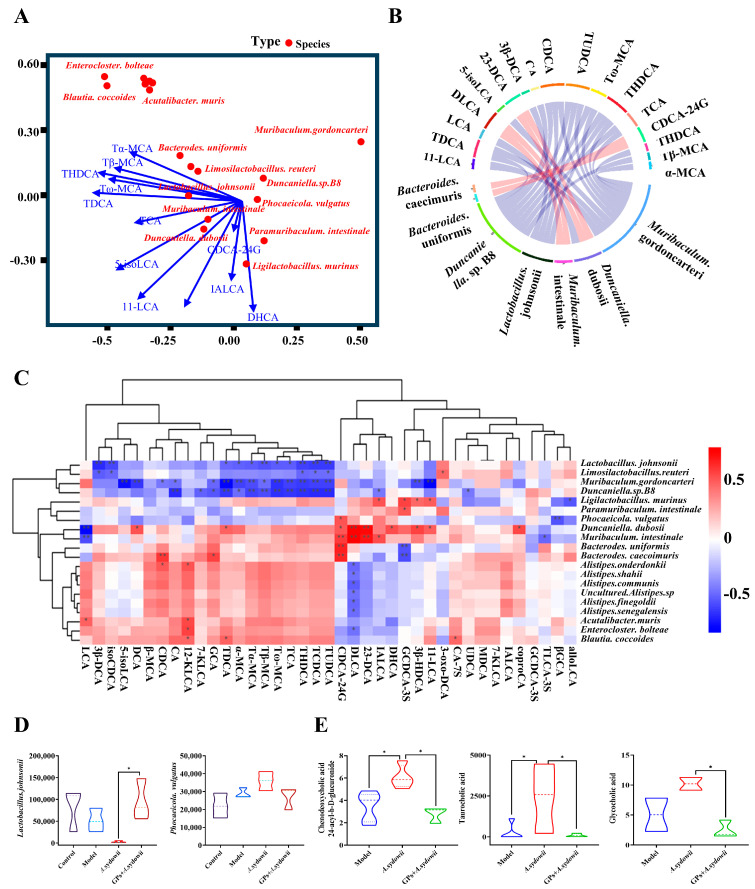
GPs suppress *A*. *sydowii*-induced tumor growth by modulating gut microbiota and bile acid metabolism. (**A**) Canonical correspondence analysis (CCA) revealed distinct clustering patterns; (**B**) Circos plot of Spearman correlations (|ρ| > 0.6, FDR < 0.05) demonstrated; (**C**) hierarchical clustering of species–metabolite correlations confirmed two distinct modules; (**D**) abundance of intestinal flora in Lewis lung carcinoma murine model; (**E**) expression levels of bile acid metabolites in Lewis lung carcinoma murine model. Data were expressed as mean ± SD. Significant comparison was determined by the Kruskal–Wallis test followed by Dunn’s multiple comparisons test (or one-way ANOVA with Dunnett’s test if the data passed normality and homogeneity tests); *n* = 5, * p < 0.05, ** p < 0.01.

An integrative analysis of metagenomic and bile acid metabolomic data was conducted to elucidate the regulatory role of GPs in gut microbiome–host co-metabolism during tumor progression. GP administration induced substantial remodeling of fecal microbiota in murine models, characterized by increased relative abundances of beneficial taxa (*Duncaniela dubosii*, *Muribaculum intestinale*) and reduced proliferation of pathogenic *Alistipes* species (*A. finegoldii*, *A. shahii*). Simultaneously, tumor tissue metabolomics showed significant reductions in DLCA and 3β-HDCA, CDCA-24G, and 5-isoLCA (Figure 5A,B). Network-based correlation analysis revealed intricate interactions between microbial taxa and bile acid metabolites. Specifically, *Ligilactobacillus. murinus* and *Duncaniella. dubosii* showed a significant positive correlation with 3β-HDCA, implying its involvement in bile acid biosynthesis. Conversely, *Muribaculum intestinale* was negatively correlated with multiple secondary bile acids, suggesting its role in bile acid metabolic transformation or regulation (Figure 5C). Furthermore, GPs treatment notably elevated the abundance of beneficial taxa such as *Lactobacillus johnsonii*, while markedly reducing the levels of pathogenic genera including *Phocaeicola. Vulgatus* (Figure 5D). GPs intervention significantly downregulated the concentrations of pro-carcinogenic bile acids (e.g., CDCA-24G, TCDCA, and GCA), which aligns with the targeted metabolomic findings (Figure 5E).

Collectively, our findings provide mechanistic insights into GP-mediated modulation of host bile acid metabolism through gut microbiota remodeling, thereby inhibiting tumor progression and attenuating *A. sydowii*-associated disease phenotypes.

### 3.6. Clinical Validation of Gut Microbiota–Bile Acid Axis Dysregulation in Lung Cancer Patients

**Figure 6:**
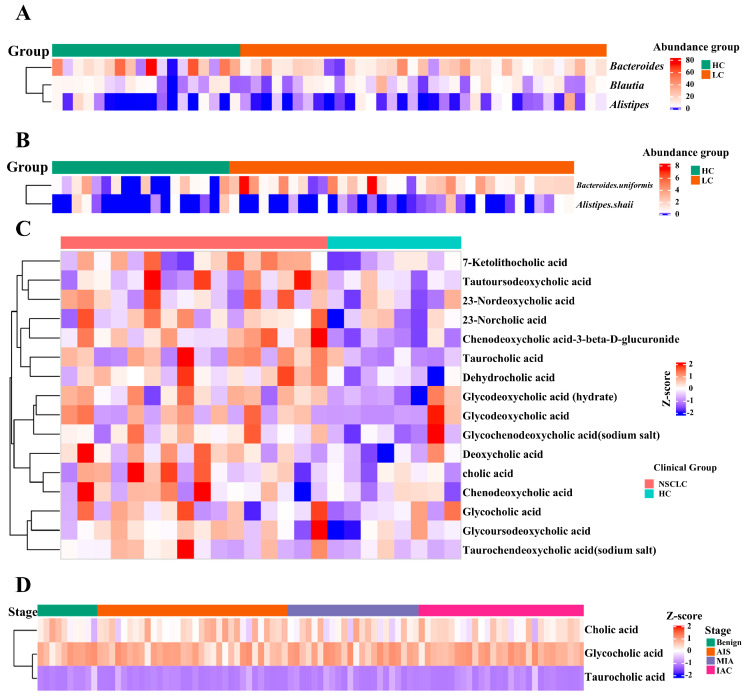
Clinical validation of gut microbiota and bile acid metabolic signatures in lung cancer patients. (**A**) Genus-level gut microbiota composition in NSCLC patients vs. healthy controls. (**B**) Species-level differential gut bacteria between NSCLC patients and controls. (**C**) Bile acid metabolite profiles in NSCLC patients vs. healthy controls. (**D**) Bile acid metabolism alterations across LUAD progression stages.

To corroborate the translational relevance of our previous findings, we conducted a cross-species validation by harmonizing clinical datasets from published cohorts. The dysregulation of gut microbiota and bile acids in patients with lung cancer was characterized. Compared to healthy controls, NSCLC patients showed a significant enrichment of pro-inflammatory genera *Alistipes Blautia* and *Bacteroides* (Figure 6A). Moreover, the species-level analysis revealed elevated *A. finegoldii* and *B. uniformis* in NSCLC, recapitulating our murine results (Figure 6B). 

Consistent with these findings, bile acid metabolomic profiles further supported our mechanistic insights. NSCLC patients displayed altered bile acid homeostasis compared to healthy individuals, with significant increases in oncogenic metabolites such as cholic acid, deoxycholic acid, glycocholic acid, etc. (Figure 6C). Longitudinal analysis across LUAD progression stages showed progressive dysregulation of bile acid metabolism, with advanced-stage tumors characterized by exacerbated cholic acid and glycocholic acid accumulation (Figure 6D). These clinical data align with our murine results, demonstrating conserved gut microbiota–bile acid axis dysregulation in lung cancer. Furthermore, the similarities between our mouse and clinical models highlight the potential of GPs as a treatment to restore this axis for managing LUAD.

## 4. Discussion

The dynamic interplay between microbial dysbiosis, metabolic reprogramming, and tumor progression has emerged as a pivotal axis in understanding cancer pathogenesis, particularly with dysregulated intratumoral mycobiomes redefining LUAD pathogenesis [17]. Accumulating evidence demonstrates that fungal colonization fosters a tumor-permissive microenvironment via β-glucan-mediated immune modulation and mycotoxin-driven genomic instability [18,19]. The present study delves into the mechanisms by which intratumoral *A. sydowii* infection, a pivotal member of the intratumoral mycobiome, accelerates tumor growth in both subcutaneous and orthotopic murine models, and examines the therapeutic potential of GPs in modulating this interaction. Our integrated metagenomic and metabolomic analyses reveal a multifaceted mechanism by which GPs reshape host–microbial interactions to attenuate pathogenic fungal-driven oncogenesis. 

One critical mechanism by which polysaccharides exert beneficial effects on LUAD is through the indirect regulation of the intestinal environmental system [20]. As key mediators of the gut–lung axis, gut microbiota influence LUAD susceptibility through metabolite-driven immunomodulation and bile acid flux regulation [21]. GPs, as a key bioactive constituent of ginseng, have been shown to positively impact LUAD through diverse mechanisms: immune modulation, induction of apoptosis, suppression of cellular proliferation/metastasis, metabolic pathway alteration, and synergistic effects with chemoradiotherapy [22,23,24]. Additionally, GPs enhance probiotic populations, restore gut barrier integrity, and mitigate inflammation [11]. Our metagenomic analysis revealed that the dysbiosis induced by *A. sydowii* infection, characterized by enrichment of pro-inflammatory taxa (such as *Alistipes finegoldii/shahii*) and depletion of beneficial commensals (e.g., *Muribaculum intestinale*), was substantially reversed by GP administration. Specifically, GPs selectively augmented beneficial taxa (e.g., *Lactobacillus*, *Muribaculum intestinale*) while suppressing pro-inflammatory *Alistipes* species (*A. finegoldii*). The enrichment of *Lactobacillus* is particularly significant, as this genus produces short-chain fatty acids (SCFAs) that modulate immune responses and inhibit tumorigenesis via histone deacetylase inhibition [25]. Conversely, the reduction in *Alistipes*, a genus associated with epithelial barrier dysfunction and pro-inflammatory cytokine secretion [26], suggests that GPs may attenuate chronic inflammation in the tumor microenvironment.

The bidirectional gut–lung axis, which integrates oropharyngeal, intestinal, and pulmonary microbiomes, plays a central role in mediating GPs’ efficacy. This bidirectional axis influences the development of both intestinal and pulmonary diseases through metabolic and immune mechanisms [27]. Central to GPs’ efficacy is the bidirectional regulation of the gut–lung metabolic axis [10]. Targeted metabolomics revealed a GP-driven shift in bile acid homeostasis, characterized by elevated 23-DCA and reduced DLCA. As an aforementioned X receptor antagonist, 23-DCA disrupts β-catenin/Wnt signaling while promoting CD8^+^ T cell infiltration [28,29]. Conversely, DLCA depletion mitigates TGR5-mediated cAMP/PKA activation linked to tumor cell proliferation [30]. Our data reveal that GPs restore bile acid homeostasis by enriching taxa involved in bile acid biotransformation, such as *Muribaculum intestinale*, thereby attenuating *A. sydowii*-induced metabolic dysregulation. Spearman correlation analysis identified *D. dubosii* as a positive regulator of 23-DCA and *M. intestinale* as a negative modulator of DLCA, quantitatively establishing microbiota–metabolite causality. These findings align with emerging evidence linking bile acid metabolism to cancer progression and highlight GPs’ ability to redirect this axis toward tumor inhibition.

Beyond the secretion of soluble metabolites, an emerging body of evidence underscores the pivotal role of microbe-derived extracellular vesicles (MEVs) as key communication materials in lung cancer pathogenesis and immunity. These nanoscale vesicles transport a diverse cargo—including proteins, lipids, nucleic acids, and even bioactive metabolites—from parental microbes to host cells, thereby modulating immune responses, inflammatory pathways, and cellular proliferation within the tumor microenvironment. It is plausible that the gut–lung axis communication facilitated by GPs may not only be mediated by microbial metabolites like bile acids but also involve the systemic delivery of immunomodulatory signals via MEVs from remodeled gut microbiota [31]. For instance, EVs from beneficial taxa enriched by GPs, such as *Lactobacillus*, could potentially contribute to the attenuation of *A. sydowii*-driven oncogenesis by promoting anti-tumor immunity, a compelling avenue for future investigation that strengthens the clinical implications of modulating the microbiome for cancer therapy.

In addition, to further substantiate our findings, we integrated published clinical datasets for cross-species analysis. Notably, the dysregulation of the gut microbiota–bile acid axis in murine models and NSCLC patients underscores its central role in LUAD pathophysiology. GPs are characterized by an increase in *Lactobacillus* and a decrease in *Alistipes*. Concomitant metabolic normalization (elevated 23-DCA, reduced DLCA) in mice mirrored healthy human profiles, demonstrating therapeutic potential. Unlike conventional antifungals limited by resistance and toxicity, GPs restore symbiotic microbiota and metabolic equilibrium for holistic therapy. These findings align with emerging strategies leveraging the microbiome–metabolome axis to improve cancer therapeutic outcomes.

Although our research offers valuable mechanistic insights, several limitations must be acknowledged. Firstly, the physiological differences between murine and human systems, such as variations in bile acid composition and immune responses, may hinder direct extrapolation of our findings. Secondly, though correlation analyses suggest a relationship between changes in microbiota and metabolites and tumor suppression, establishing causality necessitates validation through gnotobiotic models or fecal microbiota transplantation. Thirdly, the bioactive components of GPs remain to be elucidated; further investigations should focus on isolating and characterizing the immunomodulatory and metabolically active fractions. Finally, clinical trials are essential to validate the efficacy of GPs in LUAD patients and to investigate potential combinatorial approaches with immunotherapy or chemotherapy.

## 5. Conclusions

Collectively, our findings illuminate GP-mediated restoration of microbial and metabolic equilibrium as a viable strategy against *A. sydowii*-associated LUAD. By orchestrating gut–lung axis communication and bile acid flux, GPs disrupt the symbiotic relationship between fungal pathobionts and tumor progression. Further investigations should delineate temporal dynamics of microbial restitution during GP treatment and validate identified taxa–metabolite networks as predictive biomarkers, ultimately advancing personalized therapeutic paradigms for mycobiome-influenced cancers.

## Data Availability

All data and materials can be obtained from the corresponding authors upon a reasonable request.

## Supplementary Materials

The following supporting information can be downloaded at: https://www.mdpi.com/article/10.3390/cancers17193134/s1, Figure S1: Routine blood test of Lewis lung carcinoma murine model. Figure S2: Routine blood test of orthotopic LUAD murine model. Figure S3: Comprehensive microbiome study. Figure S4: Abundance of gut microbiota in Lewis lung carcinoma murine model. Figure S5: Quality control (QC) sample analysis for targeted bile acid metabolomics. Figure S6: Expression levels of bile acid metabolites in Lewis lung carcinoma murine model. Table S1: Characteristics of bile acid.

## Abbreviations

The following abbreviations are used in this manuscript:

GPs	Ginseng polysaccharides
NSCLC	Non-small-cell lung cancer
LUAD	Lung adenocarcinoma
A. *sydowii*	*Aspergillus sydowii*
LLC cells	Lewis lung carcinoma
H&E	Hematoxylin and eosin
23-DCA	Nor-deoxycholic acid
DLCA	dehydrolithocholic acid
11-LCA	Lithocholenic acid
3β-HDCA	β-Hyodeoxycholic Acid
UDCA-3S	3-Sulfo-ursodeoxycholic Acid Disodium Salt
DCA-3-O-S	Deoxycholic Acid 3-O-Sulfate Disodium Salt
HCA	Hyocholic acid
MDCA	Murideoxycholic acid
3-oxo-CA	3-Oxocholic acid
3β-UDCA	3β-Ursodeoxycholic Acid
UDCA	Ursodeoxycholic acid
HDCA	Hyodeoxycholic acid
CA	Cholic acid
ECDF	Empirical cumulative distribution function
CCA:	Canonical correspondence analysis
HC	Healthy controls
OTUs	Operational taxonomic units
PCA	Principal component analysis
