# Supplementary material for: Ginseng Polysaccharides Inhibit Aspergillus sydowii-Driven Lung Adenocarcinoma via Modulating Gut Microbiota–Bile Acid Metabolism Axis
Source: Cancers (Basel). 2025 Sep 26;17(19):3134. doi: 10.3390/cancers17193134 (PMC12524178; doi:10.3390/cancers17193134)
Supplement: Supplementary file 1 [file cancers-17-03134-s001.zip › cancers-3843462-supplementary.pdf]

cancers

Ginseng Polysaccharides Inhibit *Aspergillus Sydowii*-driven Lung Adenocarcinoma via Modulating Gut Microbiota-Bile acid Metabolism Axis

Jinlian He [1,†], Xiao Shu [2,†], HuDan Pan [2,], Mingming Wang [3], Yuanyuan Song [2], Feng Zhou [1], Lirong Lian [2], Liqing Chen [1], Gangyuan Ma [1], Yicheng Zhao [2], Runze Li [2,*] and Liang Liu [1,2,*]

1 Guangzhou National Laboratory, Guangzhou 510000, China; he_jinlian@gzlab.ac.cn (J.H.); zhou_feng@gzlab.ac.cn (F.Z.); chen_liqing@gzlab.ac.cn (L.C.); ma_gangyuan@gzlab.ac.cn (G.M.);
2 State Key Laboratory of Traditional Chinese Medicine Syndrome/Chinese Medicine, Guangdong Laboratory, The Second Affiliated Hospital of Guangzhou University of Chinese Medicine, Guangzhou 510006, Guangdong, China; 20221110736@stu.gzucm.edu.cn (X.S.); hdpan@gzucm.edu.cn (H.D.P.); songyuanyuan2017@sibcb.ac.cn (Y.S.); 20222120188@gzucm.edu.com (L.L.); yichengzhao@live.cn (Y.Z.);
3 Institute of Traditional Chinese Medicine Pharmacology, Shandong Academy of Chinese Medicine, Jinan 250014, China; 1909853dct30001@student.must.edu.mo
† These authors contributed equally to this work
* Correspondence: lirunze@gzucm.edu.cn; lirunzetk@163.com (R.L.); lliu@gzucm.edu.cn (L.L.); Tel.: +86 15363740993 (R.L.); Tel.: +86 39318472 (L.L.)

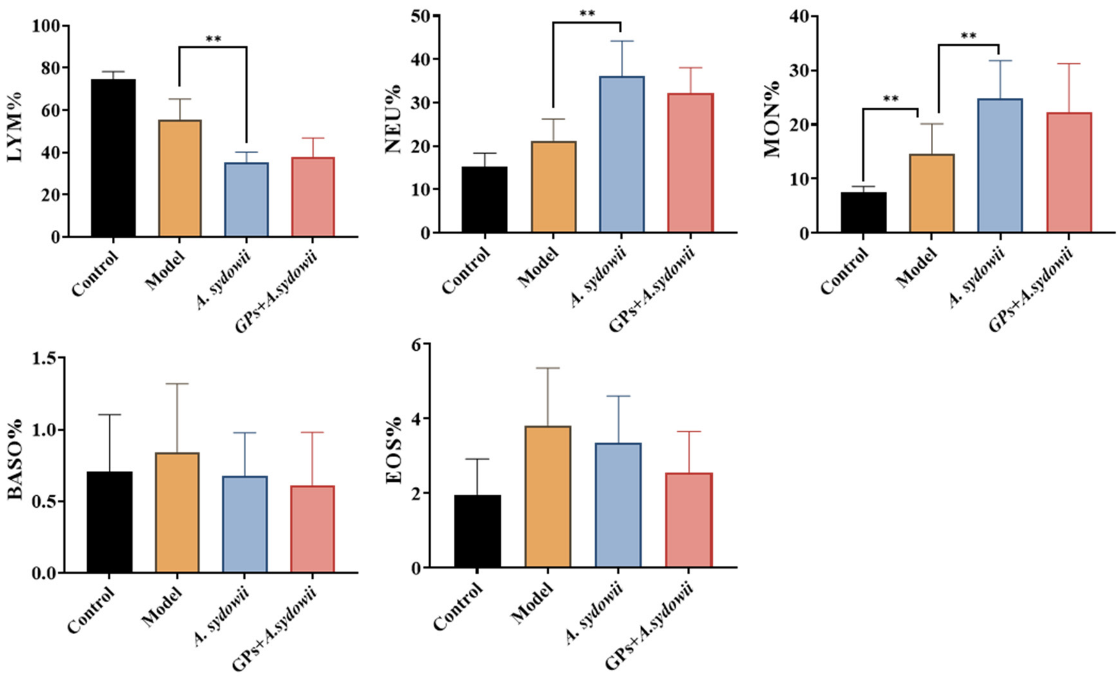

Figure S1. Routine blood test of Lewis lung carcinoma murine model. Data were expressed as mean ± SD. Significant comparison was determined by the Kruskal-Wallis test followed by Dunn's multiple comparisons test (or one-way ANOVA with Dunnett's test if the data passed normality and homogeneity tests), $n = 8$, ** $P < 0.01$ vs *A. sydowii* group.

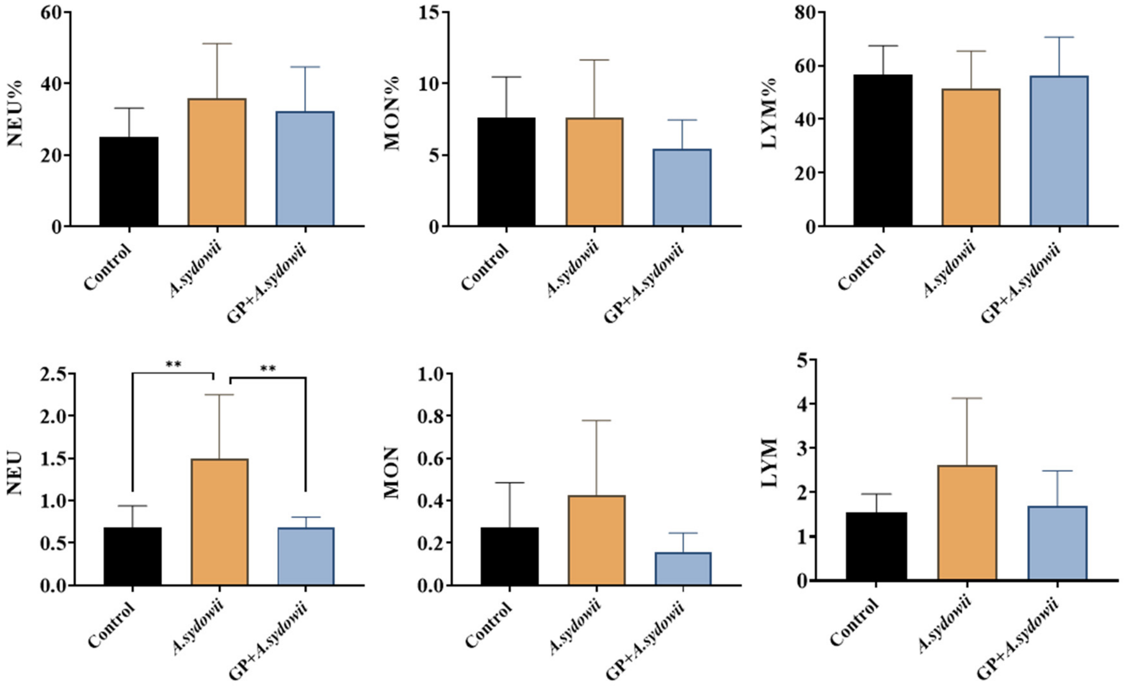

Figure S2. Routine blood test of orthotopic LUAD murine model. Data were expressed as mean ± SD. Significant comparison was determined by the Kruskal-Wallis test followed by Dunn's multiple comparisons test (or one-way ANOVA with Dunnett's test if the data passed normality and homogeneity tests), $n = 8$, ** $P < 0.01$ vs *A. sydowii* group.

A

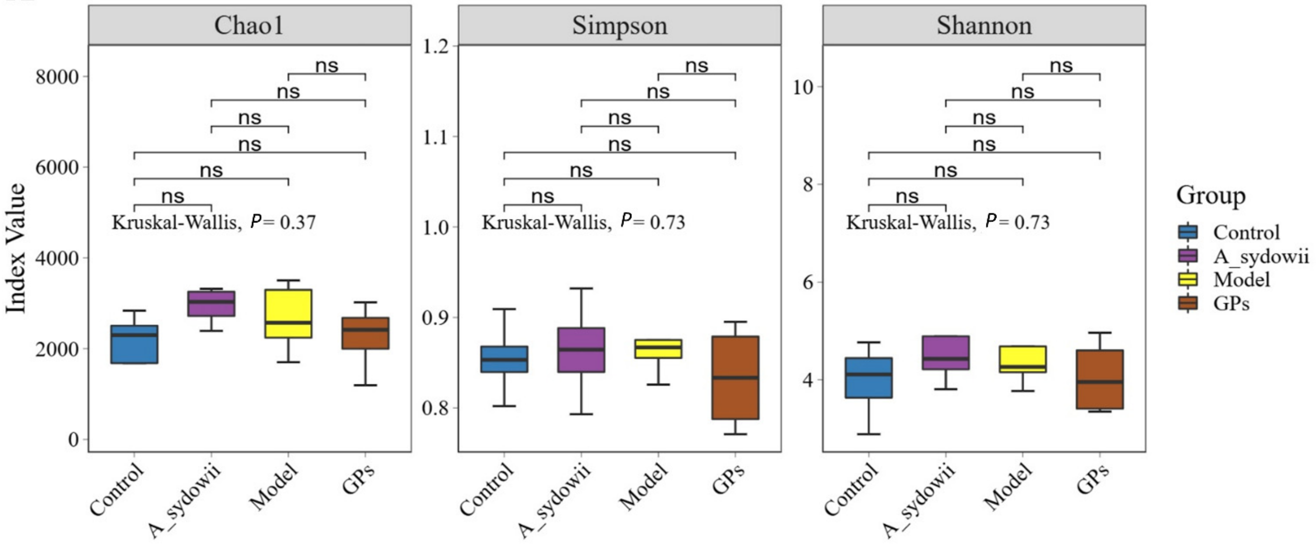

B

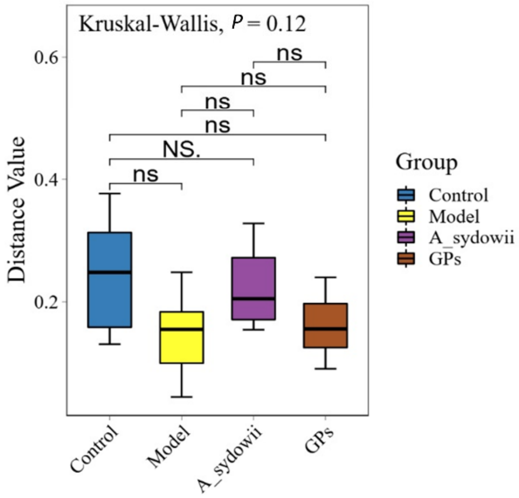

Figure S3. Comprehensive microbiome study. (**A**) alpha- diversity; (**B**) beta-diversity. Data were expressed as mean ± SD. Significant comparison was determined by the Kruskal-Wallis test followed by Dunn's multiple comparisons test (or one-way ANOVA with Dunnett's test if the data passed normality and homogeneity tests), $n = 5$.

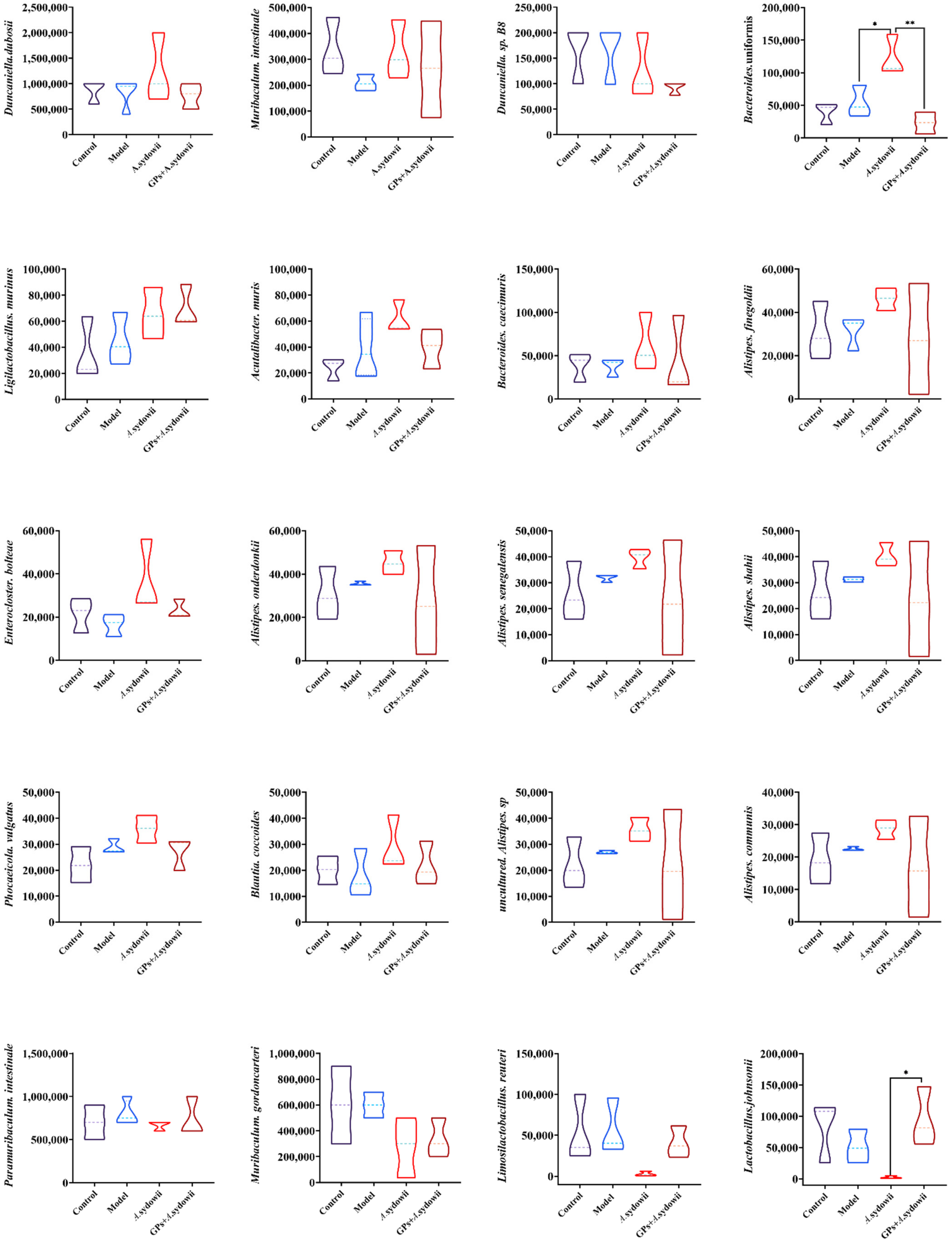
Duncaniella.dubosii
Muribaculum. intestinale
Duncaniella. sp. B8
Bacteroides.uniformis
Ligilactobacillus. murinus
Acutalibacter. muris
Bacteroides. caecimuris
Alistipes. finegoldii
Enterocloster. bolteae
Alistipes. onderdonkii
Alistipes. senegalensis
Alistipes. shahii
Phocaeicola. vulgatus
Blautia. coccoides
uncultured. Alistipes. sp
Alistipes. communis
Paramuribaculum. intestinale
Muribaculum. gordoncarteri
Limosilactobacillus. reuteri
Lactobacillus.johnsonii
Control
Model
A.sydowii
GPs+A.sydowii
*
**

Figure S4. Abundance of intestinal flora in Lewis lung carcinoma murine model. Data were expressed as mean ± SD. Significant comparison was determined by the Kruskal-Wallis test followed by Dunn's multiple comparisons test (or one-way ANOVA with Dunnett's test if the data passed normality and homogeneity tests), $n = 5$, * $P < 0.05$, ** $P < 0.01$ vs *A. sydowii* group.

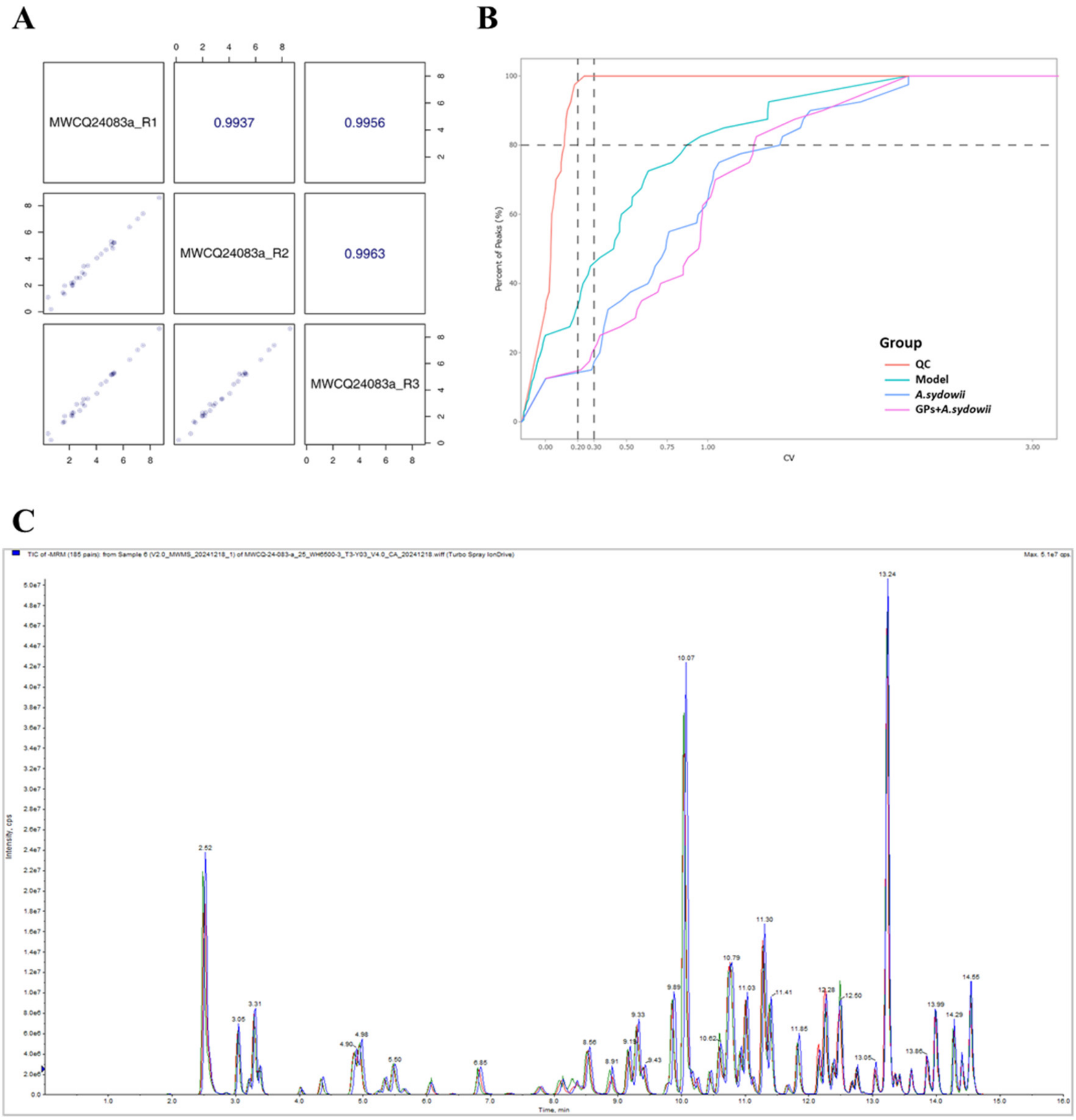

Figure S5. Quality control (QC) sample analysis for targeted bile acid metabolomics. (**A**) Pearson correlation heatmap of QC samples. (**B**) Coefficient of Variation plot of CV values for metabolites in QC samples. (**C**) Total ion chromatogram (TIC) of mixed standards.

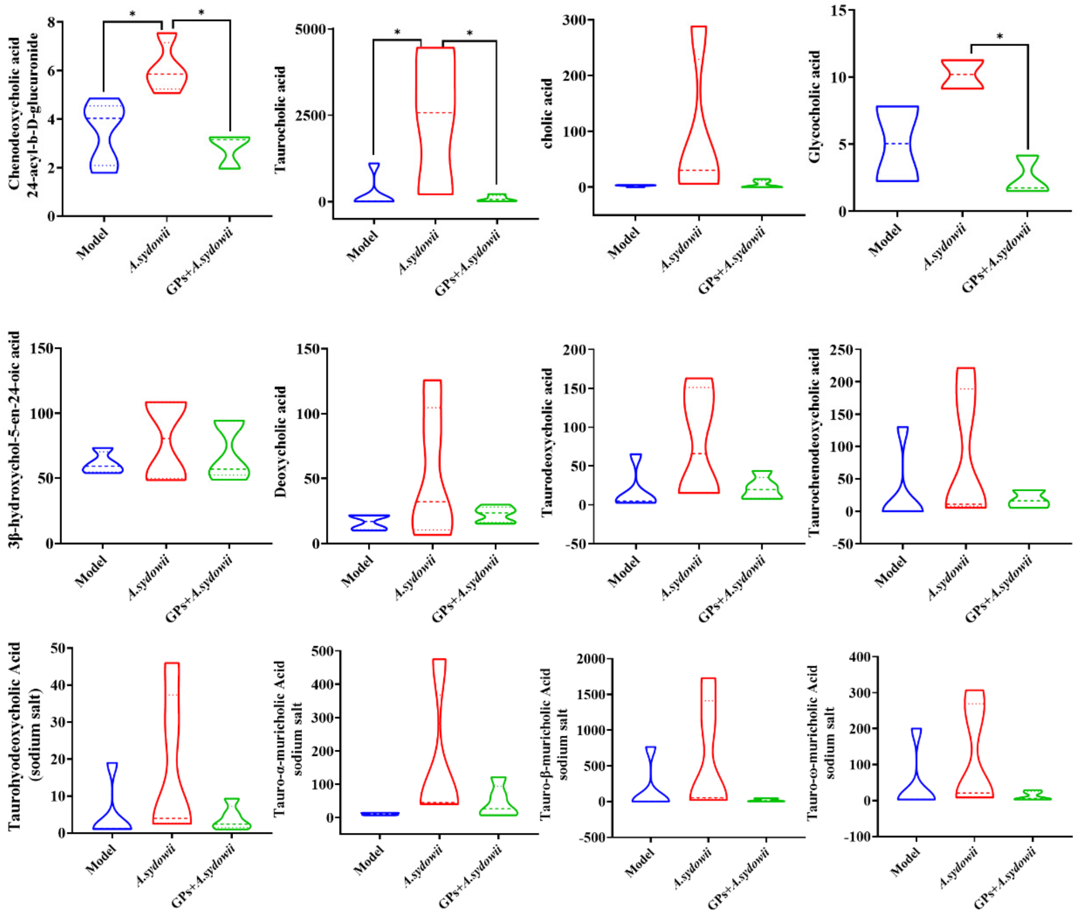

Figure S6. Expression levels of bile acid metabolites in Lewis lung carcinoma murine model. Data were expressed as mean ± SD. Significant comparison was determined by the Kruskal-Wallis test followed by Dunn's multiple comparisons test (or one-way ANOVA with Dunnett's test if the data passed normality and homogeneity tests), n = 5, * P < 0.05, ** vs *A. sydowii* group.

Table S1 Characteristic of bile acid.

Index	Compounds	Formula	Class	Retention time (min)	m/z	Molecular Weight
11-LCA	LITHOCHOLENIC ACID	C24H38O3	BAs	13.98	1.00	374.56
12-DHCA	12-dehydrocholic acid	C24H38O5	BAs	9.33	1.05	406.60
12-KLCA	12-ketolithocholic acid	C24H38O4	BAs	12.5	1.00	390.28
12-oxo-CDCA	12-Oxochenodeoxycholic acid	C24H38O5	BAs	9.33	1.00	406.27
23-DCA	Nor-Deoxycholic Acid	C23H38O4	BAs	12.4	1.00	378.28
3-oxo-CA	3-Oxocholic acid	C24H38O5	BAs	10.76	1.40	406.27
3-oxoCDCA	3-oxochenodeoxycholic acid	C24H38O4	BAs	13.24	5.64	390.60
3-oxo-DCA	3-oxodeoxycholic acid	C24H38O4	BAs	13.24	1.00	390.28
3β-CA	3β-Cholic Acid	C24H40O5	BAs	8.33	1.19	408.29
3β-DCA	3β-deoxycholic acid	C24H40O4	BAs	12.27	1.00	392.29
3β-HDCA	β-Hyodeoxycholic Acid	C24H40O4	BAs	11.03	1.00	392.29
3β-UDCA	3β-Ursodeoxycholic Acid	C24H40O4	BAs	10.81	1.00	392.29

5-isoLCA	3β-hydroxychol-5-en-24-oic acid	C24H38O3	BAs	13.61	1.00	374.60
6,7-DKLCA	6,7-diketolithocholic acid	C24H36O5	BAs	12.45	1.16	404.26
6-ketoLCA	5-β-Cholanic Acid-3α-ol-6-one	C24H38O4	BAs	11.85	1.00	390.28
7,12-DKLCA	7,12-diketolithocholic acid	C24H36O5	BAs	6.99	1.05	404.26
7-KDCA	7-Ketodeoxycholic acid	C24H38O5	BAs	9.19	1.00	406.27
7-KLCA	7-ketolithocholic acid	C24H38O4	BAs	12.29	1.00	390.28
ACA	allocholic acid	C24H40O5	BAs	8.14	1.13	408.60
alloLCA	5α-CHOLANIC ACID-3α-OL	C24H40O3	BAs	14.52	1.00	376.30
apoCA	apocholic acid	C24H38O4	BAs	12.51	1.05	390.56
CA	cholic acid	C24H40O5	BAs	10.95	1.19	408.29
CA-3G	Cholic Acid 3-O-b-Glucuronide Disodium Salt	C30H48O11	Glucuronide	4.98	7.76	584.70
CA-3S	Cholic Acid 3 Sulfate Sodium Salt	C24H40O8S	BAs	6.85	1.00	488.24
CA-7S	cholic acid 7 sulfate	C24H40O8S	BAs	6.08	1.00	488.24
CDCA	Chenodeoxycholic acid	C24H40O4	BAs	13.06	1.00	392.29
CDCA-24G	Chenodeoxycholic acid 24-acyl-b-D-glucuronide	C30H48O10	Glucuronide	8.39	3.24	568.70
CDCA-3,7S	Chenodeoxycholic acid 3,7-disulfate trisodium salt	C24H37Na3O10S2	BAs	4.87	1.17	552.70
CDCA-3Gln	Chenodeoxycholic acid-3-β-D-glucuronide	C30H48O10	Glucuronide	8.15	1.00	568.32
CDCA-3S	chenodeoxycholic acid3-sulfate disodium salt	C24H40O7S	BAs	9.89	1.00	472.25
coproCA	Trihydroxycholestanoic Acid	C27H46O5	BAs	13.39	1.00	450.70
DCA	Deoxycholic acid	C24H40O4	BAs	13.21	1.13	392.29
DCA-3,12S	Deoxycholic acid 3,12-disulfate trisodium salt	C24H37Na3O10S2	BAs	4.88	1.17	552.70
DCA-3-O-S	Deoxycholic Acid 3-O-Sulfate Disodium Salt	C24H40O7S	BAs	10.07	1.00	472.25
DHCA	Dehydrocholic acid	C24H34O5	BAs	7.8	1.00	402.24
dioxo-CDCA	3,7-DIKETOCHOLANIC ACID	C24H36O4	BAs	12.69	1.22	388.54
dioxo-HDCA	3,6-DIKETOCHOLANIC ACID	C24H36O4	BAs	12.68	1.18	388.54
DLCA	Dehydrolithocholic acid	C24H38O3	BAs	14.55	1.00	374.28
GCA	Glycocholic acid	C26H43NO6	Glyco	5.67	6.27	465.31
GCDCA	Glycochenodeoxycholic acid	C26H43NO5	Glyco	9.3	6.06	449.31
GCDCA-3S	Glycochenodeoxycholic Acid 3 Sulfate Disodium Salt	C26H43NO8S	BAs	4.38	1.00	529.27
GDCA	Glycodeoxycholic acid	C26H43NO5	Glyco	9.81	6.07	449.31
GDHCA	Glycodehydrocholic acid	C26H37NO6	Glyco	2.86	6.20	459.26
GHCA	Glycohyocholic acid	C26H43NO6	Glyco	3.39	6.27	465.31
GHDCA	Glycohyodeoxycholic Acid	C26H43NO5	BAs	5.5	1.00	449.31
GLCA	Glycolithocholic acid	C26H43NO4	Glyco	12.27	5.84	433.32
GLCA-3S	glycolithocholic acid-3-sulfate	C26H43NO7S	Glyco	7.32	5.27	513.28

GUDCA	Glycoursodeoxycholic acid	C26H43NO5	Glyco	5.37	6.07	449.31
GUDCA-3S	Glycoursodeoxycholic Acid 3 Sulfate Sodium	C26H43NO8S	BAs	2.59	1.00	529.27
HCA	hyocholic acid	C24H40O5	BAs	10.27	1.00	408.29
HDCA	Hyodeoxycholic acid	C24H40O4	BAs	11.41	1.00	392.29
IALCA	Isoallolithocholic acid	C24H40O3	BAs	13.86	1.00	376.30
IDCA	Isodeoxycholic acid	C24H40O4	BAs	14.02	1.13	392.29
ILCA	isolithocholic acid	C24H40O3	BAs	13.99	1.00	376.30
isoCDCA	Isochenodeoxycholic Acid	C24H40O4	BAs	12.18	1.00	392.29
LCA	Lithocholic acid	C24H40O3	BAs	14.41	1.00	376.30
LCA-3G	Lithocholic Acid 3-O-Glucuronide	C30H48O9	Glucuronide	10.47	1.00	552.70
LCA-3S	lithocholic acid-3-sulfate	C24H40O6S	BAs	11.68	4.69	456.25
MDCA	murideoxycholic acid	C24H40O4	BAs	10.62	1.00	392.29
NCA	norcholic acid	C23H38O5	BAs	8.86	1.19	394.27
TCA	Taurocholic acid	C26H45NO7S	Tauro	5.27	6.44	515.29
TCA-3S	Taurocholic Acid 3 sulfate sodium salt	C26H45NO10S2	Tauro	2.53	1.00	595.25
TCDCA	Taurochenodeoxycholic acid	C26H45NO6S	Tauro	8.58	6.24	499.30
TDCA	Taurodeoxycholic acid	C26H45NO6S	Tauro	9.2	6.24	499.30
TDHCA	Taurodehydrocholic acid	C26H39NO7S	Tauro	2.64	6.36	509.24
THCA	Taurohyocholic acid	C26H45NO7S	Tauro	4.05	6.44	515.29
THDCA	Taurohyodeoxycholic Acid (sodium salt)	C26H45NO6S	Tauro	4.95	1.00	499.30
TLCA	taurolithocholic acid	C26H45NO5S	Tauro	11.14	6.03	483.30
TLCA-3S	taurolithocholic acid-3-sulfate	C26H45NO8S2	Tauro	5.55	1.00	563.26
TUCA	TAUROURSOCHOLIC ACID	C26H45NO4S	BAs	14.29	1.00	467.71
TUDCA	Tauroursodeoxycholic acid	C26H45NO6S	Tauro	4.96	6.24	499.30
TUDCA-3S	Tauroursodeoxycholic Acid-3-Sulfate Sodium Salt	C26H45NO9S2	BAs	1.97	1.25	579.73
Tα-MCA	Tauro-α-muricholicAcid sodium salt	C26H45NO7S	Tauro	3.23	1.00	515.29
Tβ-MCA	Tauro-β-muricholic acid	C26H45NO7S	Tauro	3.3	6.44	515.29
Tω-MCA	Tauro-ω-muricholic Acid sodium salt	C26H45NO7S	Tauro	3.05	1.00	515.29
UCA	Ursocholic acid	C24H40O5	BAs	6.43	1.19	408.29
UDCA	Ursodeoxycholic acid	C24H40O4	BAs	11.31	1.00	392.29
UDCA-3S	3-Sulfo-ursodeoxycholic Acid Disodium Salt	C24H40O7S	BAs	10.07	4.87	472.60
α-MCA	α-muricholic acid	C24H40O5	BAs	8.91	1.00	408.29
βGCA	3β-Glycocholic Acid	C26H43NO6	BAs	3.39	1.00	465.31
β-MCA	β-muricholic acid	C24H40O5	BAs	9.43	1.00	408.29
ω-MCA	ω-muricholic acid	C24H40O5	BAs	8.33	1.05	408.29
